# Notes on stochastic (bio)-logic gates: computing with allosteric cooperativity

**DOI:** 10.1038/srep09415

**Published:** 2015-05-15

**Authors:** Elena Agliari, Matteo Altavilla, Adriano Barra, Lorenzo Dello Schiavo, Evgeny Katz

**Affiliations:** 1 Dipartimento di Fisica, Sapienza Università di Roma, Italy; 2 Dipartimento di Matematica, Sapienza Università di Roma, Italy; 3 Department of Chemistry and Biomolecular Science, Clarkson University, New York, USA

## Abstract

Recent experimental breakthroughs have finally allowed to implement in-vitro reaction kinetics (the so called *enzyme based logic*) which code for two-inputs logic gates and mimic the stochastic AND (and NAND) as well as the stochastic OR (and NOR). This accomplishment, together with the already-known single-input gates (performing as YES and NOT), provides a logic base and paves the way to the development of powerful biotechnological devices. However, as biochemical systems are always affected by the presence of noise (e.g. thermal), standard logic is not the correct theoretical reference framework, rather we show that statistical mechanics can work for this scope: here we formulate a complete statistical mechanical description of the Monod-Wyman-Changeaux allosteric model for both single and double ligand systems, with the purpose of exploring their practical capabilities to express noisy logical operators and/or perform stochastic logical operations. Mixing statistical mechanics with logics, and testing quantitatively the resulting findings on the available biochemical data, we successfully revise the concept of cooperativity (and anti-cooperativity) for allosteric systems, with particular emphasis on its computational capabilities, the related ranges and scaling of the involved parameters and its differences with classical cooperativity (and anti-cooperativity).

Cell's life is based on a hierarchical and modular organization of interactions among its molecules^1^: a functional module is defined as a discrete ensemble of reactions whose functions are *separable* from those of other molecules. Such a separation can be of spatial origin (processes ruled by short range interactions) or of chemical origin (processes requiring specific interactions)^2^. The latter, i.e., chemical specificity, is at the basis of biological information processing^3^,^4^. A paradigmatic example of this is the signal transduction pathway of the so called *two signal model* in immunology by which an effector lymphocyte needs two signals (both integrated on its membrane's highly-specific receptors in a close temporal interval) to get active^5^: these signals are the presence of the antigen (via the complex MHC-TCR) *and* the consensus of an helper-cell (via CD40 and an eliciting cytokine); this constitutes a biological, stochastic AND gate^6^. We added the adjective *stochastic* because, quoting Germain, “as one dissects the immune system at finer and finer levels of resolution, there is actually a decreasing predictability in the behavior of any particular unit of function”, furthermore, “no individual cell requires two signals (…) rather, the probability that many cells will divide more often is increased by co-stimulation”^7^: as a result, standard logic (where operations follow a deterministic chain) plays as the *ideal* reference framework, while an operative one -a stochastic formulation of logic- should take into account the presence of noise too.

Beyond countless natural examples, biologic gates have been realized even experimentally, see e.g. Refs. 8–18, the ultimate goal being the realization of stochastic, yet controllable, biological circuits^19^,^20^,^21^,^22^.

Such striking outcomes also arouse a great theoretical attention aimed to develop a self-contained framework able to highlight their potentialities and suggest possible developments. In particular, statistical mechanics has proved to be a proper candidate tool for unveiling biological complexity: in the past two decades statistical mechanics has been applied to investigate intra-cellular (e.g. metabolomics^23^,^24^, proteinomics^25^,^26^) as well as extra-cellular (e.g. neural networks^3^,^27^, immune networks^28^,^29^) systems. Also, statistical mechanics intrinsically offers a partially-random scaffold which is the ideal setting for a stochastic logic gate theory.

Another route to unveil the spontaneous information processing capabilities of biological matters is naturally constituted by information theory (see e.g. Refs. 30, 31 and references therein): remarkably, statistical mechanics and information theory (see the seminal works by Khinchin^32^,^33^, and by Jaynes^34^,^35^) and, in turn, information theory and logics (see the seminal works by Von Neumann^36^, and by Chaitin^37^) have been highlighted to be deeply connected. Therefore, it is not surprising that even in the quantitative modeling of biological phenomena these two routes are not conflicting but, rather, complementary.

In this work, we will use the former (statistical mechanics) to describe a huge variety of biochemical allosteric reactions, and then, through the latter (mathematical logic), we will show how these reactions naturally encode stochastic versions of boolean gates and are thus capable of noisy information processing.

We will especially focus on allosteric reactions (as those of Koshland, Nemethy and Filmer (KNF)^38^ and Monod-Wyman-Changeaux (MWC)^39^) as they play a major role in enzymatic processes for which a great amount of experimental data is nowadays available. However, classical reaction kinetics (i.e. those coded by Hill, Adair, etc.^40^) can also perform logical calculations and they have been set in a statistical mechanical scaffold in Ref. 19: along the paper we will deepen the crucial differences between the two types of kinetics -*allosteric cooperativity* versus *standard cooperativity*- when framed within statistical mechanics.

## Results

In the case of allosteric receptors, several models have been introduced. Many of these assume that a receptor can exist in either an active or inactive state, and that binding of a ligand biases the receptor to one of the two states. In particular, in the Monod-Wyman-Changeaux (MWC) model, ligand-bound receptors can be in either state, but coupled receptors switch between states in synchrony. Beyond that pioneering work, several models able to provide qualitative and quantitative descriptions of binding phenomena have been further introduced in the Literature, as e.g. the sequential model by Koshland, Nemethy and Filmer (KNF).

Here we consider MWC-like kinetics, and we reformulate it into a statistical mechanical framework. We start by introducing terminology and parameters for mono-receptor/mono-ligand systems (playing for single input gates as YES and NOT) and then we expand such a scenario in order to account for the kinetics of more complex systems (double-receptors/double-ligands, as those will play for two-input gates as AND, NAND, OR, NOR).

The plan is as follows: Once introduced the microscopic settings (e.g., the occupancy states *σ_i_*, *i* ∈ (1, …, *n*) of *n* receptors and the dissociation energy *h*), we define the Hamiltonian functions *H*(*σ*, *h*) coding for the chemical bindings; then -being *β* the thermal noise *β* = 1/*k_B_T* (where *k_B_* is the Boltzmann constant and *T* represents the temperature) - we build the related Maxwell-Boltzmann probabilistic weights ∝ exp[−*βH*(*σ*, *h*)]. The latter allows computing the partition functions 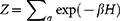, both for the active state *Z_A_* and for the inactive *Z_I_* state; the ratios, *p_A_* = *Z_A_*/(*Z_A_* + *Z_I_*) and *p_I_* = *Z_I_*/(*Z_A_* + *Z_I_*) then return the probabilities of the active/inactive states as functions of the parameters (e.g. *β*, *h*, *n*).

These probabilities are first analyzed from a logic perspective in order to show how they can account for boolean gates and then used to successfully fit the outcomes of the experiments on enzyme based logic. This route, although rather lengthy, shows why allosteric mechanisms share similar behaviors with those of classical cooperativity, but, at the same time, clearly reveals deep differences between these phenomena.

### System description

**Figure 1:**
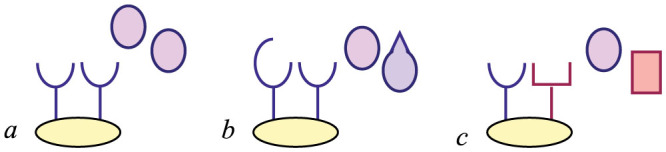
This scheme summarizes the kind of systems we are considering here: Mono-receptor/Mono-ligand (*a*), Mono-receptor/Double-ligand (*b*) and Double-receptor/Double-ligand (*c*). In this cartoon all molecules are shown as dimeric, but cases *a* and *b* also work with monomeric structures. In the Mono-receptor/Mono-ligand case only one kind of receptor and one kind of ligand (compatible with the receptor) are considered; in the Mono-receptor/Double-ligand case we still have one kind of receptor, but two different ligands both compatible with the receptor; in the Double-receptor/Double-ligand case we consider molecules displaying two different receptors in the presence of two different ligands, each compatible with only one receptor. The kinetics of these systems is addressed in *System description* Section, while in Section *Logical Operation* they are shown to work as YES, OR, and AND logic gates. See also Ref. 42.

Specifically, we start considering a system built of several molecules, each displaying one or more receptors. Each receptor exhibits multiple binding sites where a ligand can reversibly bind, and which can exist in two different states (i.e. active and inactive). In general the receptors exhibited by a given molecule can differ in e.g., the number of binding sites, the affinity with ligands, etc. As we are building a theory for single and double input gates, in the following, we will focus on simple systems where receptors can house only one or two kinds of binding sites, as exemplified in Fig. 1.

The simplest system we consider is made of a set of receptors of the same kind and in the presence of a unique ligand (see panel *a* in Fig. 1). More precisely, each receptor is constituted by *n* functionally identical binding sites indexed by *i*, whose occupancy is given by a boolean vector *σ* = {*σ_i_*}, *i* = 1, …, *n* where *σ_i_* = 1 (respectively 0) indicates that the binding site *i* is *occupied* (respectively *vacant*).

As required by the *all-or-none* MWC model, each receptor is either *active* (T) *or inactive* (R); the receptor state is indicated by a boolean *activation parameter a*, (*a* = 0, 1)^41^,^42^.

In the *absence of the ligand*, the active and inactive states (which are assumed to be in equilibrium) differ in their chemical potential, whose delta, indicated by *E*, can, in principle, be either positive (favoring the inactive state) or negative (favoring the active state): note that, the presence of a difference *E* in energy between the active and inactive states implies an exponentially unbalanced ratio between their relative concentrations (ruled by the Maxwell-Boltzmann weight).

Given a system of receptor molecules in the *absence of ligand* and in equilibrium at a given temperature *T*, we pose the following assumptions:

(a) As both the active and inactive state may coexist, the composition of the system also depends on the parameter *L* ≡ *L*(*β*) > 0, namely the *equilibrium constant* at inverse temperature *β* (in proper units, namely setting the Boltzmann constant *K_B_* ≡ 1). Letting [*R*] be the total concentration of the receptors, [*R_A_*] (respectively [*R_I_*]) the concentration of the *active* (respectively *inactive*) receptors in the *in absence of ligand*, it is [*R*] = [*R_A_*] + [*R_I_*] and [*R_A_*] = *L*[*R_I_*];

(b) For the sake of simplicity, binding sites of a mono-receptor are considered as functionally identical (as in the original model^39^).

In the *absence of ligand*, we also need to establish which of the two states (namely the *active* and *inactive* one) has a higher chemical potential. As shown in the Literature (see Ref. 41 and below) the choice is in general arbitrary (i.e. case dependent), hence we take both possibilities into account. We therefore consider two sets of *mutually exclusive* assumptions (the latter of which is denoted by a “prime” symbol).

(c) The active state has a *higher* chemical potential. Notice that, while this assumption is in contrast with the original MWC model^39^, the model itself is still self consistent as thoroughly explained in Ref. 41. The same conclusion may be drawn by the fact that, in the MWC paper, the opposite assumption is merely exploited for calculations. (i.e. *E* > 0), as e.g. in Refs. 41, 43, hence the inactive state must then be predominant (to minimize energy) (i.e. 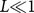);

AUT

(*c*′) The active state has a *lower* chemical potential (i.e. *E*′ ≡ −*E* < 0) as e.g. in the original MWC model^39^, hence (still for minimum energy requirement) the active state must then be predominant (i.e. 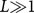).

For a thorough comparison of these two alternative assumptions (and those of the original MWC) we refer to Tab. 1.

For the sake of clarity we will from now on refer to the (c)-type assumptions as “assumptions 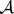
” and to the (*c*′)-type assumptions as “assumptions 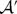
”. We also refer to the 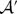 -set of assumptions as *dual* to assumptions 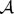, where this terminology is introduced to match the one of mathematical logic and will be therefore explained in Section “*Logical operations*”. All the assumptions without a dual one are taken to be part of both the assumption sets.

Let us now discuss the case of a system of receptor molecules in the *presence of ligand*. Clearly, the behavior of the system is expected to depend on ligand's concentration [*S*] *and* on the receptor state (i.e. either active or inactive). The dependence on the receptor state is formalized by introducing *dissociation constants K_A_* and *K_I_* for the receptor in the *active* and *inactive* state, respectively (see Ref. 42). Letting [(*R_A_S*)*_i_*] be the concentration of the receptor/ligand complex's molecules which have exactly *i* occupied binding sites, we define the average concentration of the *active* receptor/ligand complex as
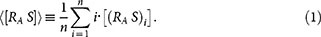
We can define the average concentration ⟨[*R_I_* *S*]⟩ of the *inactive* receptor/ligand complex in an analogous way, and we can then set
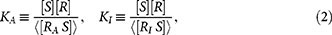
in accordance with the original presentation of MWC model (in Ref. 39, p. 90, *microscopic dissociation constants of a ligand* […] *bound to a stereospecific site are considered, whose arithmetic weighted means we denote as global dissociation constants*.). The dynamics of the receptor/ligand system is therefore determined by the variable [*S*] and the parameters *K_A_*, *K_I_*.

Now, considering both the ligand and the receptor/ligand solution we assume that

(d) receptor-ligand solution is homogeneous and isotropic. This mean-field-like assumption is actually a key assumption of all the approaches in modeling classical reaction kinetics, see e.g. Ref. 19.

Finally, a ligand can play as an activator (i.e. its presence enhances the receptor activation) as well as a suppressor (i.e. its presence hindrances receptor's activation) depending on the chemical potential time by time associated to the chemical reaction under examination (assumptions (c)'s). This choice is dual and will be deepened later: to avoid *trivial* (i.e. *static*) behavior of the system, we have to set either

(e) The ligand is an *activator*, i.e. the presence of the ligand enhances activation of the receptor. Therefore, the occupation of each receptor singularly *decreases* the energy required for activation by a parameter 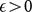.

AUT

(*e*′) The ligand is a *suppressor*, i.e. the presence of the ligand hindrances activation of the receptor. Therefore, the occupation of each receptor singularly *increases* the energy required for activation by a parameter 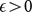.

#### Mono receptor/Mono ligand (MM) properties at equilibrium

Under assumptions 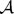, any mono-receptor/mono-ligand system, built by *n* receptors [*i* ∈ (1, …, *n*)], and whose occupancy is ruled by *σ_i_* = (0, 1), can be described by the following allosteric Hamiltonian function
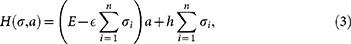
where we recall that *E* is the energy delta given by chemical potential, and *h* is the dissociation energy, namely the energy captured by a single binding site of the inactive state receptor by binding to a ligand molecule; the term in the brackets accounts for the fact that ligand acts as an activator since, for the active state (*a* = 1) binding is energetically favored, while in the inactive state (*a* = 0) the related term disappears in the Hamiltonian that reduces to the last term accounting for the bare association energy only.

By the same reasoning under assumptions 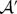, we obtain
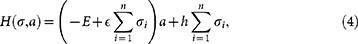


**Figure 2:**
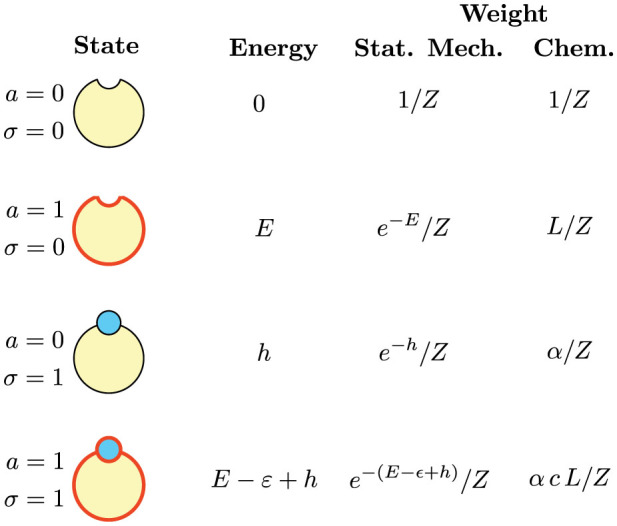
This scheme summarizes the states and the weights of the simplest MWC molecule (that, in turn, codes for the YES gate). Having only one binding site (*n* = 1), the number of possible states is four, from top to bottom: inactive and vacant, active and vacant, inactive and occupied, active and occupied. Each state corresponds to an energy, to a statistical mechanics weight and to a chemical weight. The energy is obtained by considering both the conformational degree of freedom of the molecule and the free energy of the binding process. The statistical mechanics weight is obtained according to the Boltzmann factor and the chemical weight is derived according to Tab. 1. See also Ref. 48.

The main features of the mono-receptor/mono-ligand systems described above are summarized in Fig. 2.

It is worth highlighting that the Hamiltonians (3) and (4) do not include any two-body couplings, i.e. any term 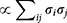: this framework is intrinsically *one-body* in the statistical mechanical vocabulary and this has implications in biochemistry too. For instance, one-body models do not undergo phase transitions and, as the latter mirror ultrasensitive reactions in chemical kinetics^19^, those are ruled out by these Hamiltonians.

Since the activation parameter is boolean, the receptor/ligand complex state may be considered regardless of the state of the receptor, by introducing the two Hamiltonians *H_A_*(*σ*) ≡ *H*(*σ*, 1) and *H_I_*(*σ*) ≡ *H*(*σ*, 0), defining the *active* and the *inactive* state energy, respectively. The corresponding partition functions are
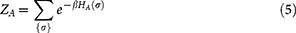

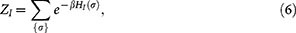
while the total partition function *Z* is given by
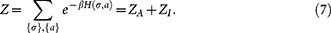
A few remarks are in order here:

– The summations in the partition function (7) account for the activation degree of freedom too. This means that the latter participate in thermalization or, in other words, that the intrinsic timescale for the dynamics of *a* is bounded from above by those of the *σ*: this is consistent with the original MWC assumptions of synchronized switches among coupled receptors (the so called *all-or-none* behavior).

– This model can be solved even *at finite n*, namely without the oversimplifying thermodynamic limit *n* → ∞.

– All the energies can be expressed in units of the thermal energy *k_B_T* ≡ *β*^−1^, hence, in order to avoid possible misunderstanding as *T* already addresses the tense molecular state and to keep notation as simple as possible, in the following we set *β* = 1, thus forcing all aforementioned parameters and variables to be dimensionless.

– As a consequence of the two previous remarks, the stochasticity is retained by the parameter *n*, such that for *n* → ∞ stochastic computing will collapse on the deterministic one (that is, classical logic) and, on the other side, the smaller *n* and the larger the noise affecting the system.

Now, focusing on *Z_A_* (as *Z_I_* is analogous), we define 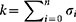, and we can therefore write
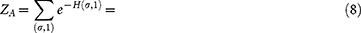

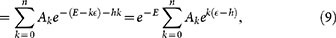
where *A_k_* denotes the number of times that the sum 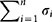 turns out to be equal to *k*. Noting that *σ* is a binary vector, we get straightforwardly that 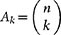, and therefore
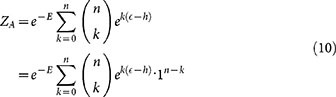

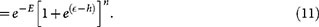
Analogously, *Z_I_* = (1 + *e*^−*h*^)*^n^*.

Thus, the probabilities *p_A_* and *p_I_* for the complex to be in the active or in the inactive state, respectively, are
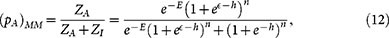

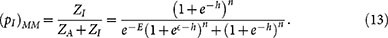
where the subscript *MM* stands for “Mono-Mono”.

Correspondingly,
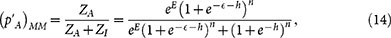

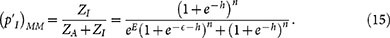


The interesting quantity to look at is (*p_A_*)*_MM_*, as it corresponds to the concentration 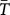 of receptors in the active state and this is expected to continuously increase (respectively decrease) with the percentage of activation enhancement (i.e. *e*^−*h*^, see Tab. 1) under assumptions 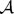 (respectively 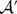). We notice, though, that the original model^39^ is concerned with 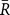 (i.e. with *p_I_*) rather than 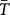; anyhow, *p_A_* and *p_I_* carry the same information as they are complementary probabilities.

Notably, the correspondence stated in Tab. 1 confirms the consequences of assumptions (c) and (e), that is, choosing *E* > 0 yields *L* < 1, while choosing 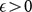 yields *c* > 1. In particular, according to the notation of Ref. 39, we have
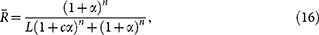

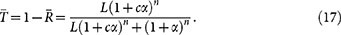
Conclusions on the dual assumptions 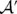 are much the same and will not be repeated.

#### Mono-receptor/Double-ligand (MD) properties at equilibrium

Under the assumptions of the previous section, any mono-receptor/double-ligand system, built by *n* receptors [*i* ∈ (1, …, *n*)] and whose occupancy is ruled by *σ_i_* = (0, 1), can be described by the following allosteric Hamiltonian function
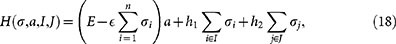
where, in contrast with the previous case described by eq. (3), two distinct ligands, whose dissociation energies are denoted by *h*_1_ and *h*_2_ respectively, are considered. More precisely, *I* and *J* are two subsets of {1, …, *n*} such that 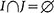, and they denote the sites linked to the first ligand and to the second ligand, respectively. To express this in formulae, we impose that 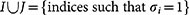.

As we did for the Mono-Mono case, the partition function coupled to the Hamiltonian (18) is given by
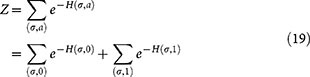

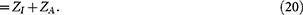
We focus on *Z_A_*, as *Z_I_* is analogous. Let us pose *k*_1_ = |*I*| and *k*_2_ = |*J*|, notice that 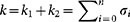, and write the sums explicitly as
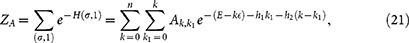
where 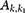 denotes the number of times that the sum 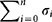 is equal to *k*, with the condition that *k*_1_ of the *σ_i_*'s belong to the set *I*. This quantity is rather tricky to calculate but can actually be rewritten in terms of multinomial coefficient (which counts the number of ways we can choose *k* elements among *n*, with the condition that they are divided in groups of *k_j_* elements each). Then, we get
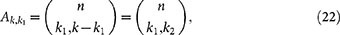
in such a way that *Z_A_* can be rewritten (using *k*_1_ and *k*_2_) as
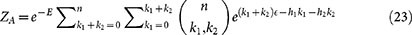

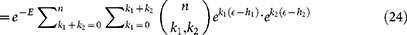

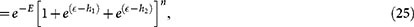
where in the second passage we must consider a 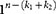 factor, which allows us to conclude the calculation, by simply expanding the trinomial.

Analogously, we obtain 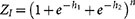.

Indeed, we have
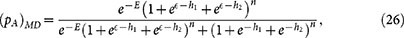

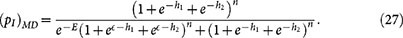


In a similar fashion, under assumptions 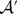, we obtain
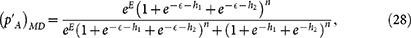

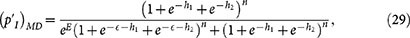
where the subscript *MD* stands for “Mono-Double”.

#### Double-receptor/Double-ligand (DD) properties at equilibrium

Under the same assumptions of the previous sections, any double-receptor/double-ligand system, built by *n* receptors [*i* ∈ (1, …, *n*)] and whose occupancy is ruled by *σ_i_* = (0, 1), and *τ_i_* = (0, 1) can be described by the following allosteric Hamiltonian function
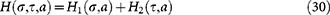

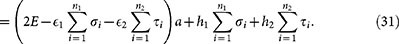
We note that the system factorizes into two independent Mono-Mono Hamiltonians, hence we can entirely skip the calculations, referring to results already presented in the section devoted to Mono-receptors/Mono-ligand properties. Thus, focusing on a symmetric case for simplicity, i.e. 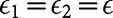 and *n*_1_ = *n*_2_ = *n*, we get
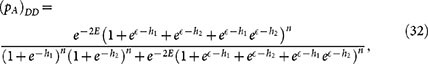
while, via the dual assumptions 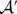, we have
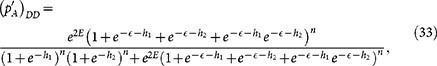
where the subscript *DD* stands for “Double-Double”.

### Logical operations

Let us now explore the possibility of using these allosteric receptor-ligand systems as operators mimicking stochastic logic gates: the *presence* of ligands (*variables* in Logic) is denoted as *S_i_* for the *i*-th ligand, and the *presence* of receptors (*operators* in Logic) is denoted as *R_A_*_,*i*_ and *R_I_*_,*i*_ for the active and inactive state of the *i*-th receptor, respectively: note that *σ_i_* and *S_i_* are conceptually different because, in Logic, *S_i_* mirrors the presence of the *i*-th ligand, that is *S_i_* = “true” stands for a high concentration presence of the *i*-th ligand, thus within the statistical mechanical route the *S*'s are linked to the *h*'s rather than to the *σ*'s.

Operators are of two kinds: the *unary* operators YES and NOT, which evaluate a single argument, and the *binary* operators, e.g., AND and OR, which evaluate two arguments.

Let us describe the examples of concrete interest in the paper:

– *Affirmation*: “S”, namely the evaluation of the *presence* of ligand *S*, which returns true if and only if the ligand *S* is present. Hereafter this operator will be denoted as stochastic YES (or, in case a distinction between several ligands is necessary, as YES*_S_*).

– *Negation*: “
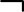
*S*”, namely the evaluation of the *absence* of ligand *S*, which returns true if and only if the ligand *S* is *not* present. Hereafter this operator will be denoted as stochastic NOT (or NOT*_S_*).

– *Conjunction*: “
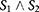
”, namely the evaluation of the presence of *both* ligands, which returns “true” whenever both ligands occur to be present (i.e., in the case that *S*_1_ and *S*_2_ are assigned value “true”) and “false” whenever at least one of the two ligands is not present (i.e., in the case that either *S*_1_ or *S*_2_ are assigned value false). The evaluation of such operator is hereafter denoted as *S*_1_ AND *S*_2_ (stochastic AND).

– *Non-exclusive disjunction*: 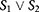, namely the evaluation of the presence of at least one ligand, which returns true whenever at least one ligand is present and value false whenever they are both absent. The evaluation of such operator is hereafter denoted as *S*_1_ OR *S*_2_ (stochastic OR).

As we will see, the receptor molecule plays as an operator, while ligands play as variables. In order to evaluate the formula, each variable can assume value either “true” of “false” according to the ligand concentration, where “true” means that the ligand is present at a concentration larger than a threshold value, while “false” means that the ligand concentration is smaller than such a value. Moreover, the value arising from the evaluation of the operators corresponds to the activation state of the receptor: active if the evaluation returns “true” and inactive is evaluation returns “false”.

#### Mono-receptor/Mono-ligand system: YES and NOT functions

All the plots in this and in the following sections are based on some scaling assumptions that will be discussed further in the paper (see Section “*Methods*”). These assumptions are essential to our purpose (that is, they enable us to tune the free variables introduced defining the Hamiltonians), and are deduced by physical and biochemical reasoning. We will refer to these assumptions as they are reported in Section *Methods* below.

**Figure 3:**
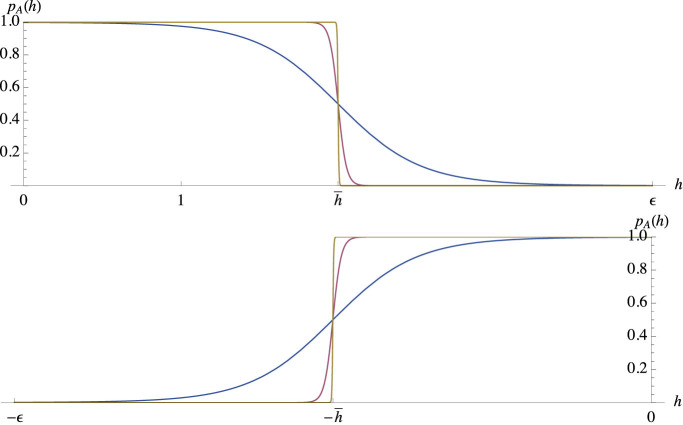
Upper panel: Sigmoidal behavior of *p_A_*(*h*) with parameters *E* = 2*n*, 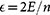, where *n* = 5 (*blue*), *n* = 50 (*red*), *n* = 500 (*gold*). Lower panel: Anti-sigmoidal behavior of 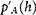 with parameters *E* = 2*n*, 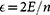, where *n* = 5 (*blue*), *n* = 50 (*red*), *n* = 500 (*gold*).

Under scaling assumptions (35), (39) and (40), plots of the activation probability (*p_A_*)*_MM_*(*h*) from eq. (12) show marked sigmoidal behavior (see Fig. 3, upper panel), signaling activation of the receptor in significative presence of the ligand, i.e. for small values of the variable *h*: the logarithmic relation between *h* and the concentration follows directly both from the original MWC model, as summarized in Table 1 and from the Thompson approach^41^.

Thus, the function (*p_A_*)*_MM_* may be considered as mimicking the logical YES_[*L*]_ function, assuming boolean values 0 for low ligand concentration and 1 for high ligand concentration, as one can see from Tab. 2.

The threshold value is set at 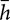 which can in turn be fixed by properly choosing the system constituents (e.g. the number of binding sites hosted by a receptor).

On the contrary, the function 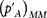 of eq. (14) may be considered as mimicking the logical NOT_[*L*]_ function (Fig. 3, lower panel), assuming boolean values 0 for high ligand concentration and 1 for low ligand concentration, as one can see from Tab. 2.

**Figure 4:**
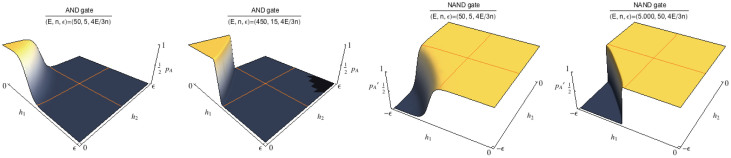
Left: (*p_A_*)*_DD_*(*h*_1_, *h*_2_) plots. Activation of the receptor is verified by small values of *h*_1_ and *h*_2_, corresponding to a significative presence of both the two ligands, thus simulating a stochastic AND function. Right: 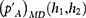 plots. Activation of the receptor is verified by high (i.e. small in absolute value) values of *h*_1_ or *h*_2_, corresponding to a significative presence of any of the two ligands, thus simulating a stochastic NAND function.

A comparison of the theoretical versus real behavior of these gates is presented in Fig. 4, while the best fitting procedure is discussed in Section “*Best fitting procedures*”.

#### Mono-receptor/Double-ligand system: OR and NOR functions

**Figure 5:**
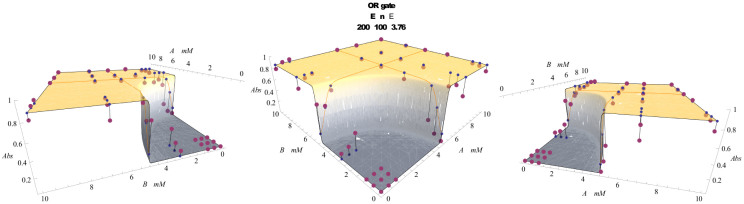
The stochastic OR gate has been realized in two coupled steps involving enzymatic processes as sketched in Fig. 7: first enzyme is esterase, that reacts with ethyl butyrate ([A]) or methyl butyrate ([B]), or both, catalyzing production of ethanol and methanol, respectively. Butyric acid is a byproduct of the process. To set the gate, the physical zeros of the signals have been fixed experimentally to convenient input values (ethyl butyrate 10 mM and methyl butyrate 10 mM). Bullets represent experimental data^8^, whereas the surface represents the best fitting according to eq. (26).

The activation probability (*p_A_*)*_MD_* (eq. (26)) can be used to model a stochastic version of the logic gate OR. In fact, if we look at the presence of the two different ligands as a binary input, the behavior of (*p_A_*)*_MD_* (with the scaling assumptions of eqs. (35), (39)), as a function of *h*_1_ and *h*_2_ (see Fig. 5), recovers the OR's one (see Tab. 2). Similarly to the YES case, the value 0 for *h*_1_ and for *h*_2_ denotes the saturation of the ligand. Therefore, consistently with the structure of OR, the presence of only one out of the two ligands is sufficient to make the molecule active; conversely, the value 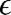 denotes the absence, thus for 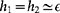, (*p_A_*)*_MD_* is vanishing, namely, it returns as output “false”. Note that the projection of the plot over 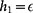 (or 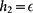) gives a sigmoid, consistently with the fact that, if one of the two inputs is constantly false, the OR recovers the YES.

Performing the same calculations, the dual counterpart 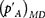 of eq. (28) models the logical NOR gate, that is the direct negation of the previous one, as shown in Fig. 5).

**Figure 6:**
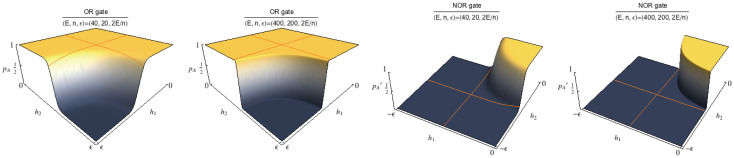
Left: (*p_A_*)*_MD_*(*h*_1_, *h*_2_) plots. Activation of the receptor is achieved by small values of *h*_1_ or *h*_2_, corresponding to a significative presence of any of the two ligands, thus simulating a stochastic OR function. Right: 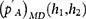 plots. Activation of the receptor is verified by small values of *h*_1_ or *h*_2_, corresponding to a significative presence of any of the two ligands, thus simulating a stochastic NOR function. Note that for smaller *n* curves are smooth (noisy), while for large *n* quasi-discontinuous jumps appear.

**Figure 7:**
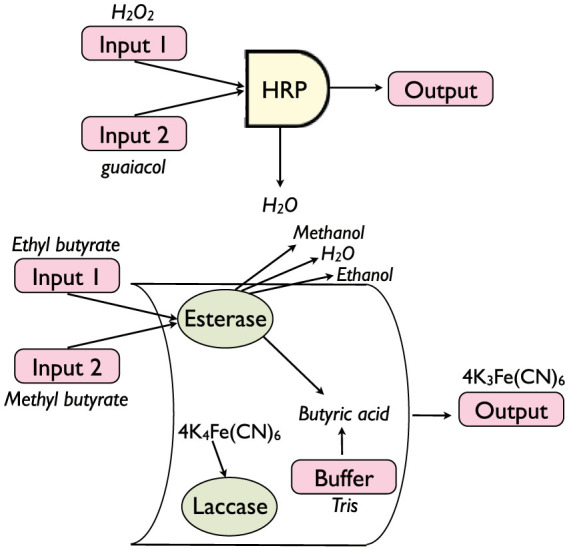
Schematic representation of the gates from a biochemical perspective. Upper panel: The stochastic AND gate is shown as a biocatalytic process. The two inputs are H_2_O_2_ and one out of three chromogens (ABTS, ferrocyanide, guaiacol) -only the latter is illustrated-. Signal processing is biocatalyzed by HRP and the output measure optically as the amount of the oxidized chromogen. See Ref. 9 for more details. Lower panel: The stochastic OR gate is shown. It involves two enzymatic processes and a buffering part. The first enzyme is esterase, that reacts with ethyl butyrate or methyl butyrate (or both) biocatalyzing production of ethanol and methanol, respectively. Butyric acid is a byproduct of the process and, as its production lowers the *pH* of the system, further a buffer is added. The product of the process is measured by absorbance at *?* = 420 nm using a UV-2401PC/2501PC UV-visible spectrophotometer with a TCC-240A temperature controller holder. See Ref. 8 for more details.

A comparison of the theoretical versus real behavior of these gates is presented in Fig. 6 (for the OR only), whose data have been collected from an experiment sketched in Fig. 7 (lower panel); the best fitting procedure is discussed in the Section “*Best fitting procedures*”.

#### Double-receptor/Double-ligand system: AND and NAND functions

**Figure 8:**
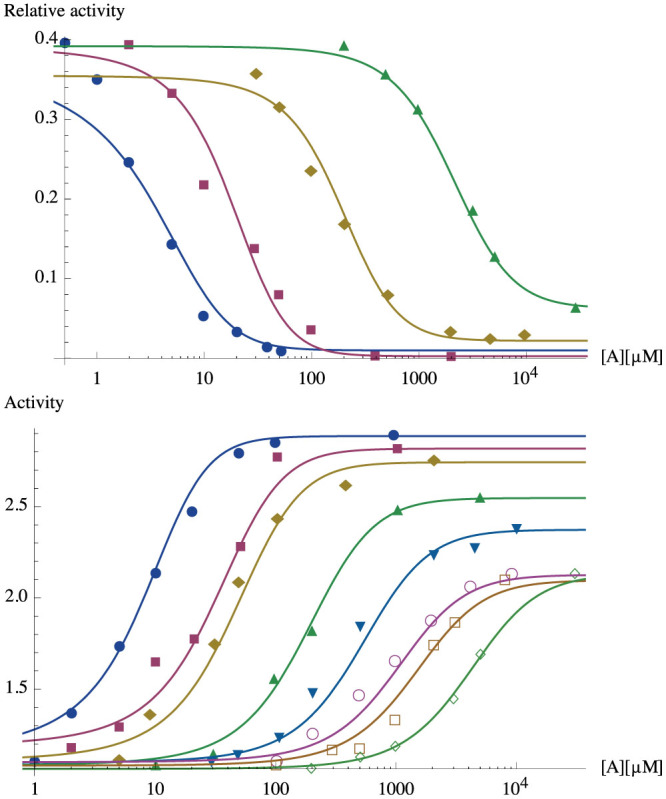
Upper panel: stochastic YES gate, achieved through the statistical mechanical formulation of the allosteric monoreceptor-monoligand complex under assumptions 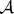 and tested on *E. colii* chemotaxis network response measured by fluorescence resonance energy transfer (FRET) to decreasing concentrations (in mM) of *?*-methylaspartate (MeAsp, [A]); data from Ref. 44. Lower panel: stochastic NOT gate, achieved under assumptions 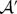 and tested on *E. colii* FRET-measured chemotaxis network response to increasing concentrations (in mM) of MeAsp ([A]); data from Ref. 45. See Refs. 44, 45 for more details.

The function (*p_A_*)*_DD_* previously described (eq. (32)) models a stochastic version of the logic AND gate (see Tab. 2). As in the case of OR, we look at the two ligands as a binary input, and we assume the scaling assumptions coded in eqs. (35), (42), (44). The resulting behavior of (*p_A_*)*_DD_* fits the one expected for the AND function, with fitness to the expected plot that sensibly improves in the extremal regions of the plot, i.e. for 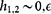 (see Fig. 8). Again, its projection returns a sigmoid because if one of the two inputs is constantly true, the AND recovers the YES.

The dual version 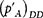 (eq. (33)) models the logic gate NAND, i.e. the direct negation of the previous one. As this negation is precisely dual, so is the shape of the plot (see Fig. 8).

**Figure 9:**
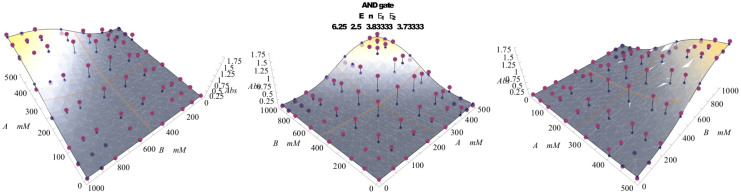
The stochastic AND gate has been realized by two inputs constituted by H_2_O_2_ ([A]) and guaiacol ([B]) as the chromogen, while l-ascorbic acid [Asc](0) = 120 *?*M was used for filtering; the signal processing was biocatalyzed by HRP, as sketched in Figure 7 (upper panel). The output was measured optically as the amount of the oxidized chromogen. Bullets represent experimental data Ref. 9, whereas the surface represents the best fitting according to eq. (32).

A comparison of the theoretical versus real behavior of these gates is presented in Fig. 9 (for the AND only), while, mirroring the exposition of the OR gate, Fig. 7 (upper panel) summarizes the experiment working as data source; the best fitting procedure is discussed in the Section “*Best fitting procedures*”.

## Conclusions

Chemical computing uses molecular systems to perform logical operations, mimicking processes typical of electronic devices. Advantages and disadvantages appear when comparing these two approaches to computation: chemical computing requires (for a single operation) a smaller size (Angstroms versus microns) and a lower energy consumption (~10^−19^ Joule versus ~ 10^−9^ Joule), yet it is slower than electronic computing (from kilohertz to megaherz versus gigahertz). Further, biochemical information processing performs at relatively large levels of noise (and this happens at different scales, ranging from enzyme-based logic gates^10^ to nucleic acid logic circuits^21^: noise propagates in the system as thermal disorder or in form of cross-talk among system's constituents^4^,^49^. Hence, classical (i.e. deterministic) logic works only as an ideal reference framework and the field of research would strongly benefit from a robust stochastic reformulation of logic gates whose properties can be safely tested and used as guides in in-vitro experiments.

In this work we showed that statistical mechanics can play a major role to accomplish this task: through statistical-mechanical models, we analyzed paradigmatic allosteric reaction kinetics and we proved their ability in performing noisy operations (hence working as suitable versions of stochastic logic gates). Moreover, we tested their performances (as resulting from the theory) on real data taken from enzyme-based information processing systems finding overall very good agreement between theoretical predictions and experimental behaviors. From this perspective, we showed that allosteric kinetics of mono receptor systems naturally encodes a noisy version of the input-output relations typical of the YES and NOT gates, while allosteric kinetics of double receptors systems plays as a stochastic version of the OR (NOR) and AND (NAND) gates. Finally, we checked that in the noiseless limit of the theory (for *n* → ∞, or, alternatively, for *β* → ∞) these gates recover the pure logic-like behavior (i.e. deterministic information processing).

The framework developed here allows establishing controllable, explicit relations among the system parameters, with remarkable practical outcomes. For instance, the noise that generally affects biological gates can be quantified permitting the management of large devices where different gates work in cascade and where noise-driven errors (possibly amplified by the various gates) actually constitute severe limitations. Also, in order to build large biochemical circuits, understanding how simple building blocks behave and how their interactions scale up with system' size is mandatory as well as quantifying the computational power of the system itself: classical reaction equations (i.e. systems of differential equations) can not accomplish these tasks, while statistical mechanics can. In fact, combining Hamiltonians coding for various gates in a cascade fashion is straightforward in the present formalization, further, an arsenal of tools stemmed from the neural network branch (e.g., methods to address system's computational capacity, memory storage, etc.^27^) becomes immediately handily via this route.

## Methods

In this section we discuss three major aspects of our work: the scaling assumptions, the role of *allosteric cooperativity* within the model and the best fitting procedures.

### Scaling assumptions

As the assumption sets 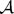 and 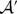 only affect the sign of the parameters *E*, 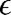 and of the variable *h*, we cannot expect every choice of these quantities to yield a realistic behavior from a biophysical viewpoint. Particularly an effective range of the variable *h* as well as some reasonable scaling properties for *E* and 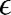 are to be determined, most likely depending on *n*.

The first issue can be solved independently of the case considered (*MM*, *MD*, *DD*). As evidenced in Tab. 1, for assumptions 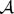 it is *e*^−*h*^ = [*S*]/*K_I_* and, being *h* positive, activation enhancement [*S*]/*K_I_* is dimensionless and ranging in [0, 1], thus, it may be considered as a *percent molar concentration* of the ligand *S*. Also, we expect that there exists a numerical (percent) value for the ligand concentration, below which the receptor activity is unaffected (see e.g. Ref. 46). We refer to this threshold value as *τ* and, according to Tab. 1, this also determines the significance range of *h* as
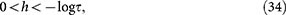
which reliably limits the range of the dissociation energy to finite values. As *τ* determines the receptor sensitivity with respect to its activity, it is reasonably expected that *τ* ≈ *K_A_*/*K_I_*; in fact such inverse proportional dependence of *τ* with respect to *K_I_* is consistent with increasing monotonicity of *h* with respect to *K_I_* (consistently with assumptions (c), (e)).

Moreover, from Tab. 1, 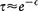, whence a reliable significance range for *h* is
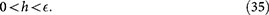


Dually, for assumptions 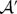 it is *e^h^* = *K_I_*/[*S*] and the same conclusion follows that *K_I_*/[*S*] may be considered as a *percent molar concentration* of the ligand *S*. As for *τ*′ we have *τ*′ ≈ *K_I_*/*K_A_* (following from assumptions (*c*′), (*e*′)), yielding
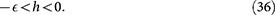


Now we focus on the scaling of *E* and 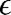: in the following we address this matter separately for the case of one or two receptors, which have different nature.

#### Mono-receptor case: YES/NOT and OR/NOR gates

We refer only to assumptions 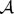, since dual gates clearly scale in the same way. Let us start considering the Mono-Mono case: given Eq. 12, we can define 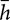 as the value of the dissociation energy such that 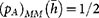, which implies
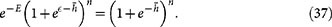


On the other hand, the active (*a* = 1) and saturated (*σ* = 1) state is an extremal state of the system corresponding to minimum entropy. As a result
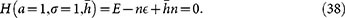


From the previous two equations we have
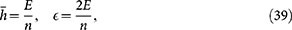


The same conclusion can be drawn independently following another route: according to the constraint (35), the maximum value attainable by the Hamiltonian (3) is *E* and it corresponds to an active state with 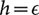; on the other hand, the minimum value attainable is 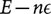, corresponding to *h* = 0 and a fully occupied state. Imposing the range interval for the energy 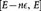 to be symmetric around 0 it must then be 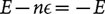, namely 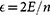. Finally we observe that *E* depends only on the receptor, therefore in the presence of a single receptor-type it must be *E* ∝ *n* in view of the linear extensivity of thermodynamics; direct verification shows that
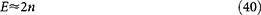
best fits our purpose.

Scaling assumptions for the OR gate are derived imposing that the behavior of the function (*p_A_*)*_MD_* recovers that of (*p_A_*)*_MM_* when one of the ligands is absent (that is, when *h*_2_ → ∞). If we carry out the calculation, we find that
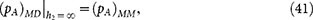
so the scaling for *E* and 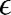 must be the same of the previous one in order to be consistent.

#### Double-receptor case: AND/NAND gates

This case differs from the Mono-receptor one mostly because of the non-linear scaling of *E*: since the receptors are dimeric, their response must be linear with respect to each functional monomer; consequently *E* ∝ *n*^2^, and again we see directly that the proper scaling is achieved by
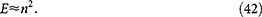


As far as the scaling of 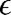 is concerned, we proceed in the same way as we did for the OR gate, and argue that posing *h*_2_ = 0 (strong presence of one ligand) must logically recover the behavior of (*p_A_*)*_MM_* from (*p_A_*)*_DD_*. In this case, however, we do not find an exact identity, but we can rearrange the result to look like


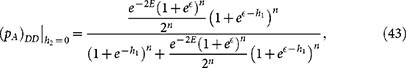
so, setting 
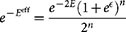
 and 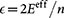 we obtain
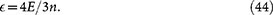


### The role of allosteric cooperativity

Now we want to deepen where the differences between the classical cooperativity and the MWC-like one, known in the Literature as *allosteric cooperativity* (see e.g., Refs. 38, 47), reside. This difference can be investigated directly from a mathematical and logical point of view by comparing the plots of the AND gate and of the OR gate.

#### OR gate: classical cooperativity

Here we discuss why and how the OR gate, that can be handled by a one-body statistical mechanical Hamiltonian (eq. (18)), does manifest a (roughly standard) cooperative behavior. The OR Hamiltonian is indeed a rigged one-body expression: cooperativity (meant as produced by a term ~ *Jσσ*, see eq. (45)) is nested within the definition of the OR Hamiltonian coded in eq. (18), hidden inside the request 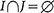. It is in fact possible to infer from this constraint that, in order to obtain the correct ensemble *K* of the indices of the occupied binding sites, it is alternatively possible to introduce two subsets *I*′ and *J*′ where only the condition *I*′, *J*′ ⊂ *N* is left to be respected: the price to pay for this simplification, however, is in writing the ensemble *K* as 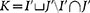, instead of 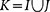. Such way of writing the OR constraints (which is nothing but a reformulation of the Inclusion-Exclusion Principle) makes explicit the presence of the cooperative term which turns out to be exactly 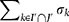. The latter can be rewritten as 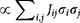 (for some positive coupling *J*) because *σ_i_σ_j_* = 1 if and only if both *σ_i_* = 1 and *σ_j_* = 1. As a further check of the latter statement it is to be noticed that the presence of a quadratic growth term accounting for proper cooperativity may be deduced by the circular edge of the upper plateau (Fig. 4), that is missing when looking at the AND gate (Fig. 5).

#### AND gate: allosteric cooperativity

In a real cooperative system there is a mutual enhancement of the activation probability; conversely, the AND gate lacks such a mutual enhancement, and the presence itself of both the ligands is simply necessary for activation, or, in other words, it is possible to (biochemically) realize an AND gate only when a (significant, that is *at high concentration*) amount of both ligands is present, independently of the percent concentration relative to any of them. Since the AND Hamiltonian (eq. (30)) results only from the juxtaposition of two YES Hamiltonians, it is truly one-body: this fact is fully consistent with the linear edge of the upper plateau in the AND plot (Fig. 5).

Note that, if instead of an allosteric mechanics (hence with the activation parameter *a* and with two different conformational states *R*, *T*), we adopted a classical (i.e. not-allosteric) cooperative Hamiltonian for the system, we would write
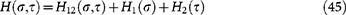

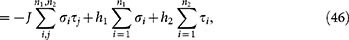
where *J* is a scalar parameter tuning the reciprocal enhancement.

Comparing eq. (30) and eq. (45) we see that they would be equivalent if we could write 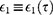 and 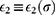 but, as log 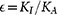, then 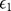 and 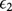 are constant dependent on the species making up the system but independent of their bounding state, that is, 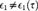 as well as 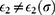 (see Ref. 19 for classical cooperativity). Therefore, we cannot express the Hamiltonian (30) as a two-body system, and this codes for the *allosteric* nature of this gate.

We perform now a brief mathematical analysis of the above mentioned shape of the AND plot (from here on referred to as a “*cut*”): a simple calculation shows that 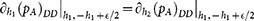, which states that the *cut* is in fact corresponding to the straight line 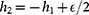 (the symmetric angular coefficient simply recalls the choice 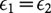). Furthermore, it is possible to prove that the slope *m* of the line projection on the *h*_1_, *h*_2_-plane is in fact 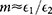. It follows that the case 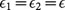 is the one best fitting the expected plot of the logical operator. On the contrary, by taking limits for either 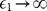 or 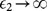, one recovers the YES_2_ (respectively YES_1_) as a projection on the (orthogonal) axis.

### Best fitting procedures

We can finally discuss how to test the predictions of the theory over the *in vitro* experiments carried on both single-input and two-inputs (bio)-logic gates: while plots summarizing our findings have already been presented, see Figures 6; 8; 9 for the reaction kinetics and Fig. 7 for a sketch of the experimental settings, here we discuss how best fits of the theory versus the data have been obtained.

Since the variable *h* and parameters *n*, *E*, 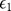, 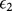 are dimensionless, any linear rescaling of the function *p_A_* is allowed (whose choice is discussed below).

#### Unary operators

In the YES case (data from Ref. 44), the opportune *y*-rescaling is obtained for each data set *D_k_* by considering the function 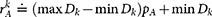. In order to compensate the logarithmical progression of the axis, the *x*-rescaling (which is effectively linear, but conveyed on a log scale) is of the form 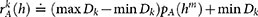 where *m* = *m_k_* is opportunely depending on *k*. The displayed function is 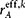, which is the same as 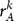, but varying the parameters *n*, 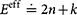, 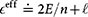. Consistently with scaling equations (39), (40), *k* varies within ±3.4%*E* and *ℓ* within 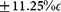.

In the NOT case (data from Ref. 45) the opportune *y*-rescaling is obtained by plotting precisely the function 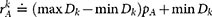 with the same *x*-rescaling as in the YES case. In order to show how precise the fitting is (*after* suitable log-lin rescaling), the best fit is obtained by considering 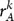 as a function of *n* = *n_k_* only, while *E_k_* = 2*n_k_* and 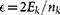, according to the assumptions, thus the fit is practically achieved with one degree of freedom only.

We emphasize that, in both cases, the fit may be improved by data extrapolation of maximal (minimal) values for the range of 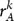 which are strictly higher (lower) than the maxima (minima) of *D_k_*.

#### Binary operators

Given the *x*_1_*x*_2_-data grid –0, …, *M*_1_} × –0, …, *M*_2_}, a (vertical) *y*-rescaling is required in order to match 1 with the experimental maximum value of the activation parameter. In order to determine such value, a *stable data set S* is opportunely defined; letting ⟨*S_z_*⟩ be the mean *z*-value of the stable data set, we take it as a reliable value for the maximal experimental activation. The opportune *y*-rescaling is therefore obtained by considering the function 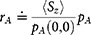, while the *x*_1_*x*_2_-rescaling is achieved by plotting 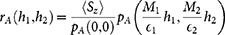.

In the OR case, the *stable data set* is taken to be the data set in the [8, 10] mM × [8, 10] mM region. The best fit is obtained by varying the parameters *n*, *k* and *ℓ*, where the plotted function is an *effective r_A_* function defined as 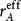, a function of *n*, 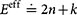, 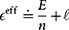. Consistently with scaling equations (39), (40), *k* varies within ±1%*E* and *ℓ* within 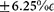.

In the AND case, the *stable data set* is taken to be the data set in the [400, 500] mM × [800, 1000] mM region. The best fit is obtained by varying parameters *n*, *k*, *ℓ*_1_ and *ℓ*_2_, where the plotted function is an *effective r_A_* function defined as 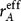, a function of *n*, 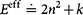, 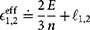. Consistently with scaling equations (42), (44), *k* varies within ±3%*E* and *ℓ*_1,2_ within 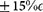.

Results are shown in Fig. 9, for the stochastic AND, and Fig. 6 for the stochastic OR.

## Figures and Tables

**Table 1:** Correspondences with the parameters originally used in Ref. 39

Stat-Mech 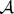 -set	Ref. 42	Ref. 39	MWC meaning	Stat-Mech, 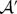 -set	Ref. 42	Ref. 39	MWC meaning
*e*^−*E*^	*ω*_0_	*L*	equilibrium constant of active/inactive–state receptor system in absence of the ligand	*e*^−*E*′^	*ω*_0_	*L*	equilibrium constant of active/inactive-state receptor system in absence of the ligand
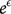	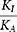	*c*	dissociation constants ratio	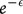	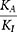	*c*^−1^	(inverse) dissociation constants ratio
*e*^−*h*^	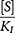	*α*	neat percentage activation enhancement	*e^h^*	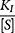	*α*^−1^	inverse neat percentage activation enhancement
*p_I_*	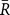		probability of the inactive (*relaxed*) state, i.e. average concentration of the receptor in the inactive state	*p_I_*	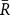		probability of the inactive (*relaxed*) state, i.e. average concentration of the receptor in the inactive state
*p_A_*	*p_A_*	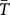	probability of the active (*tense*) state, i.e. average concentration of the receptor in the active state	*p_A_*	*p_A_*	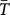	probability of the active (*tense*) state, i.e. average concentration of the receptor in the active state

**Table 2:** The truth table of all the logical operators introduced by now

Input		YES*_A_*	NOT*_A_*	*A* OR *B*	*A* NOR *B*	*A* AND *B*	*A* NAND *B*
A	B	*A*	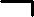 *A*	*A* ∨ *B*	*A* ↓ *B*	*A* ∧ *B*	*A* ↑ *B*
1	1	1	0	1	0	1	0
1	0	1	0	1	0	0	1
0	1	0	1	1	0	0	1
0	0	0	1	0	1	0	1
